# Supplementary material for: Reliability of a Custom Device Used to Measure Isometric Knee Flexor and Extensor Strength in Standing Position
Source: Life (Basel). 2023 Feb 6;13(2):458. doi: 10.3390/life13020458 (PMC9965042; doi:10.3390/life13020458)
Supplement: Supplementary file 1 [file life-13-00458-s001.zip › life-2118143-supplementary/S1.pdf]

Universal load cell **CVS**

available with certification • EAC

General information

PVS15920210224

The universal CVS load cell, built in stainless steel and designed for force measurements, is able to work both in traction and in compression and has excellent characteristics of linearity, precision and resistance. In fact, it withstands overloads of over 300% and has waterproof protection for use in severe environmental conditions. Furthermore, the load cell is equipped with an M8-PG7 cable gland and a 5-meter shielded cable with 4 conductors.

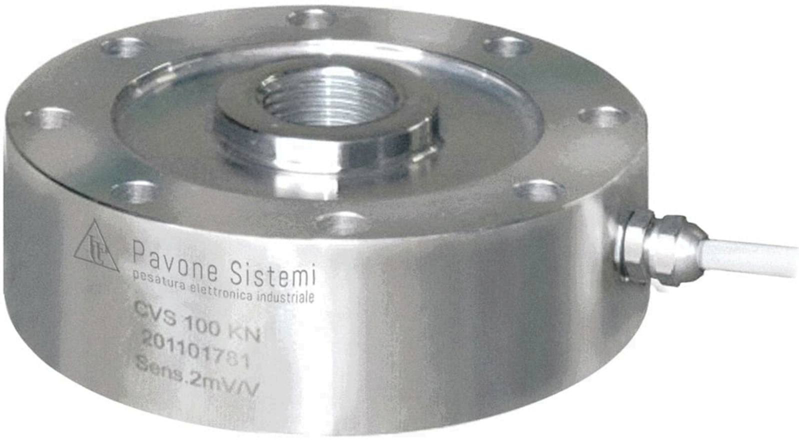

Suggested related products

A highly performing weighing system must be accurate, perfectly calibrated and well maintained. In order to improve the load cell performance and to optimize its functioning, you may need the following products:

Weight Transmitter UWT 6008

Weight Transmitter DAT 1400

Compression load cell CC3

Universal load cell CVF

Double Shear Beam Load Cell DDR

All indicated data may be changed without notice.
All the measures indicated are expressed in millimeters (mm).

PAVONE SISTEMI S.R.L.
Via Tiberio Bianchi 11/12/13, 20863 Concorezzo (MB), Milan, Italy
T (+39) 039 9162656 **F** (+39) 039 9162675 **W** pavonesistemi.com
Industrial Electronic Weighing Systems since 1963

Technical specifications

PVS15920210224

**Rated load RL:**	5, 10, 20, 30, 50, 100, 200, 300, 600, 1000 kN
**Combined error:**	±0.08 % RO
**Repeatability:**	±0,02 % RO
**Creep (20 minutes):**	±0,02 % RO
**Safe overload:**	150 % RL
**Ultimate overload:**	> 300 % RL
**Material:**	Stainless steel AISI 17-4PH
**Degree of protection:**	IP67
**Deflection:**	0.35 mm
**Compensated Temperature:**	-20 ÷ +70 °C
**Temperature range:**	-10 ÷ +50 °C
**Temperature effect on zero balance:**	< ±0.005 % RO/°C
**Temperature effect on output:**	< ±0.005 % RO/°C
**Rated output RO:**	2.0 mV/V ±0.1 %
**Zero balance:**	< ±1.5 % RO
**Insulation resistance:**	> 5000 MOhm
**Input resistance:**	700 ±20 Ohm
**Output resistance:**	700 ±5 Ohm
**Recommended input:**	2 ÷ 15 Vdc/ca

All indicated data may be changed without notice.
All the measures indicated are expressed in millimeters (mm).

PAVONE SISTEMI S.R.L.
Via Tiberio Bianchi 11/12/13, 20863 Concorezzo (MB), Milan, Italy
T (+39) 039 9162656 **F** (+39) 039 9162675 **W** pavonesistemi.com
Industrial Electronic Weighing Systems since 1963

Universal load cell **CVS**

available with certification • EAC

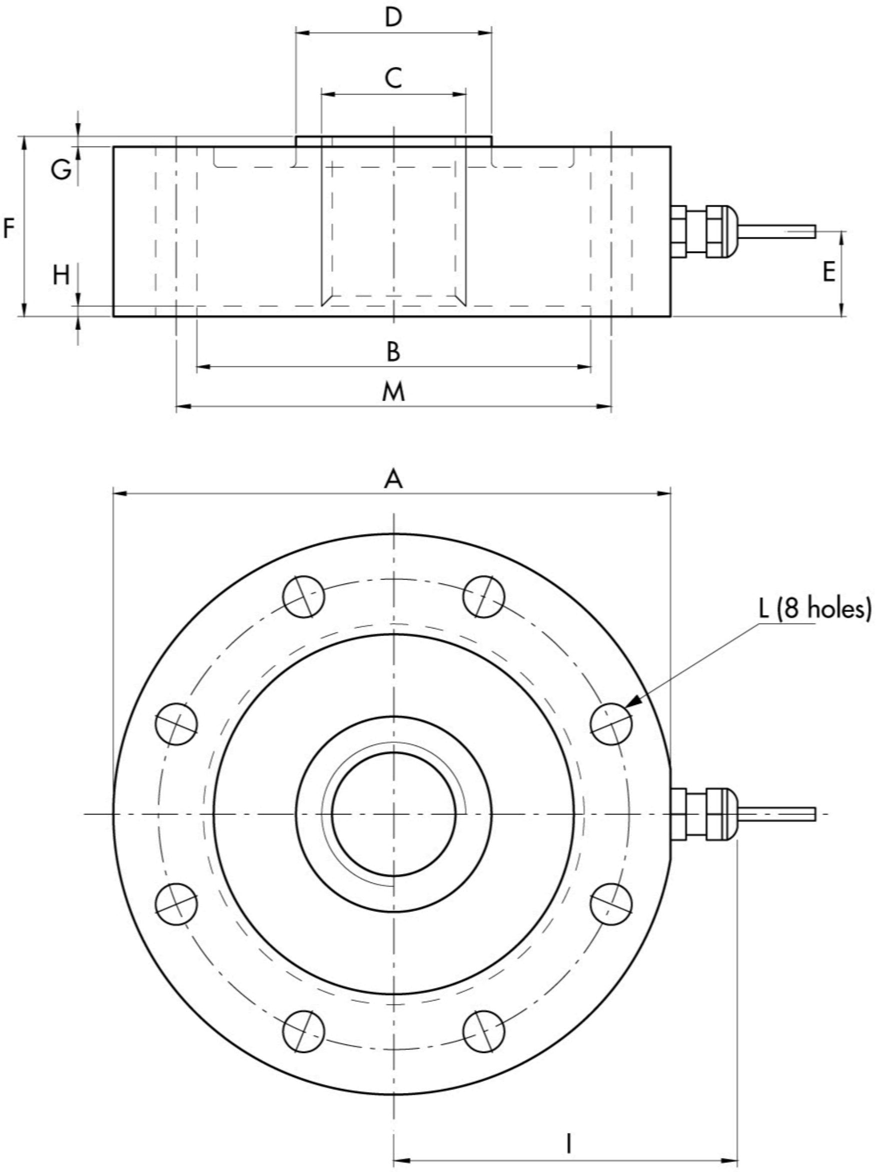

Capacity kN	ØA	ØB	ØC	ØD	E	F	G	H	I	ØL	M
5-30	99	72.2	M20x1.5	31	14	30	2	1.5	60	8.5	85
50-100	109	78.3	M24x2	38	15	35	2	1.5	65	8.5	94
200-600	164	113	M48x3	70	13.5	50	5	2	100	16.5	136
1000	219	135	M64x4	88	19	70	5	3	120	26	175

Electrical Connections	
+Excitation	Red
-Excitation	Black
+Sense	Blue
-Sense	Brown
+Signal	Green
-Signal	White
Shield	Cable shield

All indicated data may be changed without notice.
All the measures indicated are expressed in millimeters (mm).

PAVONE SISTEMI S.R.L.
Via Tiberio Bianchi 11/12/13, 20863 Concorezzo (MB), Milan, Italy
T (+39) 039 9162656 **F** (+39) 039 9162675 **W** pavonesistemi.com
Industrial Electronic Weighing Systems since 1963
